# Four-Dimensional Printed Construct from Temperature-Responsive Self-Folding Feedstock for Pharmaceutical Applications with Machine Learning Modeling

**DOI:** 10.3390/pharmaceutics15041266

**Published:** 2023-04-18

**Authors:** Purushottam Suryavanshi, Jiawei Wang, Ishaan Duggal, Mohammed Maniruzzaman, Subham Banerjee

**Affiliations:** 1 Department of Pharmaceutics, National Institute of Pharmaceutical Education & Research (NIPER)-Guwahati, Changsari 781101, Assam, India; 2 Pharmaceutical Engineering and 3D Printing Lab (PharmE3D), Division of Molecular Pharmaceutics and Drug Delivery, College of Pharmacy, The University of Texas at Austin, Austin, TX 78712, USA

**Keywords:** 4D construct, smart materials, machine learning, drug delivery

## Abstract

Four-dimensional (4D) printing, as a newly evolving technology to formulate drug delivery devices, displays distinctive advantages that can autonomously monitor drug release according to the actual physiological circumstances. In this work, we reported our earlier synthesized novel thermo-responsive self-folding feedstock for possible SSE-mediated 3D printing to form a 4D printed construct deploying machine learning (ML) modeling to determine its shape recovery behavior followed by its potential drug delivery applications. Therefore, in the present study, we converted our earlier synthesized temperature-responsive self-folding (both placebo and drug-loaded) feedstock into 4D printed constructs using SSE-mediated 3D printing technology. Further, the shape memory programming of the printed 4D construct was achieved at 50 °C followed by shape fixation at 4 °C. The shape recovery was achieved at 37 °C, and the obtained data were used to train and ML algorithms for batch optimization. The optimized batch showed a shape recovery ratio of 97.41. Further, the optimized batch was used for the drug delivery application using paracetamol (PCM) as a model drug. The % entrapment efficiency of the PCM-loaded 4D construct was found to be 98.11 ± 1.5%. In addition, the *in vitro* release of PCM from this programmed 4D printed construct confirms temperature-responsive shrinkage/swelling properties via releasing almost 100% ± 4.19 of PCM within 4.0 h. at gastric pH medium. In summary, the proposed 4D printing strategy pioneers the paradigm that can independently control drug release with respect to the actual physiological environment.

## 1. Introduction

Three-dimensional (3D) printing technology or additive manufacturing is a ground-breaking technology used in the medical industry for applications such as drug delivery systems or devices, tissue or organ fabrication, and regenerative medicines [1]. Further, this technology customizes patient-specific drug delivery devices according to the patient’s need [2]. However, the majority of the pharmaceutical-grade materials printed using 3D printing techniques are static and unable to change in response to the physiological environment. To overcome this problem, the biomaterials should adapt to the dynamic physiological conditions over time [1]. The technology of 4D printing has emerged to solve this problem.

The term 4D printing was first coined by Tibbits in 2013 in a TED talk to emphasize “time” as a novel aspect for developing 3D entities [3]. To this end, 4D printing was illustrated to demand either biomaterial that can transform from one programmed structure to another or multi-material prints that can transform over time, thus providing static movement to the 3D object [3]. Later, in its use by other scientists, the term 4D printing was extended to the targeted development of 3D entities in terms of shape, properties, and functionality. Finally, 4D printing gained its true meaning by referring to the preparation of objects by various 3D printing technologies using smart materials as a feedstock, depicting self-transformation abilities in response to the dynamic physiological environment [3,4,5]. Stimulus-responsive biomaterials or polymers appeared to hold particular promise for such smart drug delivery systems due to their ability to show a reversible change in physical and chemical properties in response to physiological conditions [6]. Among various stimuli-responsive polymeric materials, the use of temperature-sensitive polymers for drug delivery is much-admired as one of the most actively explored stimuli-responsive materials [7,8]. The most commonly used temperature-responsive shape memory polymers (SMPs) are limited to poly(lactic acid), and thermoplastic polyurethane (TPU), where Rahmatabadi et al. [9] investigated the microscopic concept of SMPs using a novel encapsulated bilayer structure of non-shape memory thermoplastic polycaprolactone (PCL)-TPU, where the thermoplastic PCL was responsible for fixing the temporary shape. Soleyman et al. [10] studied polyethylene terephthalate glycol (PET-G) as a new temperature-responsive SMP. The examined SMP showed excellent shape memory effect with 96% of shape recovery.

Among the range of thermosensitive polymers, PNIPAM is regarded as one of the most popular materials [6,8,11]. PNIPAM shows a reversible coil-to-globule phase transition at its lower critical solution temperature (LCST) of 32 °C in an aqueous environment, a unique property in the physiological respect [8,12]. Due to its unique property, PNIPAM has already been explored for cell culturing, drug delivery, and directed protein adsorption, the intelligent actuator [13,14]. In particular, PNIPAM containing block co-polymers can release the medicament in response to the temperature. Further, the modifications in the PNIPAM chain with hydrophobic and hydrophilic moiety can modulate encapsulation/release behavior [15].

The development of novel strategies for the controlled delivery of drugs or cells in the human body is considered of prime importance for medicines and regenerative therapy. In this regard, considering the novelty of 4D printing in developing controlled drug delivery systems is yet to be fully explored. Thus, the objective of the present work was to prepare temperature-responsive self-folding (both placebo and drug-loaded) feedstock into a 4D printed construct using SSE-mediated 3D printing technology. Here we have used p(NIPAM-4-ABP)-PCL gel at different ratios without and with the addition of PCM (as a model anti-pyretic drug) to investigate the shape memory behavior and drug release capabilities of the 4D construct. The prepared feedstock was evaluated for rheological properties, whereas the printed 4D construct was further evaluated for temperature-responsive shape memory behavior followed by drug encapsulation and drug release investigations. In this regard, the parameters, such as shape fixity and shape recovery ratio, were calculated for different batches, and the obtained data were used to train the ML algorithm. Both the DT and the MLR equation were used to optimize the best shape recovery and shape fixity behavior of the optimal formulation. Further, the optimized formulation through such an ML approach was systemically evaluated to investigate the 4D construct solid-state properties using Raman spectroscopy, DSC, TGA, and XRD analysis. Additionally, the morphological inspection of the 4D construct before and after shape recovery was assessed using SEM. Finally, the PCM-loaded 4D construct was programmed into a temporary shape and evaluated for its *in vitro* PCM delivery capability in an acidic buffer.

## 2. Materials and Methods

### 2.1. Materials

Poly(caprolactone) (PCL, (C_6_H_10_O_2_)n) was purchased from Sigma Aldrich, Co., Spruce Street, St. Louis, MO, USA. PCM (C_8_H_9_NO_2_) was purchased from Sigma Aldrich, Co., Spruce Street, St. Louis, MO, USA. PNIPAM-4-Acryloyloxybenzophenone (4-ABP, C_16_H_12_O_3_) was synthesized using the same materials as mentioned in our earlier publication [16]. The other reagents and solvents were purchased of analytical grade and used as received.

### 2.2. Preparation of Temperature-Responsive Self-Folding Placebo Feedstock

Thermo-responsive self-folding p(NIPAM-4-ABP) was synthesized as per the procedure mentioned in our earlier publication [16], followed by the preparation of temperature-responsive self-folding placebo feedstock with and without addition of polycaprolactone (PCL) at different concentrations (P1–P6) as shown in Table 1. The prepared different ratios blend was stirred on a magnetic stirrer at 60 °C for 30 min in order to make the desired printing feedstock for 3D printing purposes.

### 2.3. SSE 3D Printing

The 3D bioprinter (INVIVO, ROKIT Healthcare, Seoul, Republic of Korea) equipped with pressure assisted syringe and UV lamp was used to print both the prepared placebo and PCM-loaded temperature-responsive self-folding feedstock, which was loaded into the disposable syringe and inserted into the cartridge for printing. The most characteristic feature for SSE 3D printing is that the material is in a semi-solid state, with the nozzle diameter affecting the printing resolution. Printing temperature is another important characteristic for SSE 3D printing, as viscosity of the gel is directly proportional to the temperature. Further, the distance between the nozzle head and the print bed is also considered an important parameter in SSE; if the distance is too large, the extruded material may accumulate in the nozzle head and not stick to the build plate properly [17]. Considering all these characteristics of SSE, the printing parameters, such as nozzle size, layer height, print speed, retraction speed and distance, and input flow were controlled using NewCreatorK software (version 1.57.71). The CAD software (Solidworks, 2019) was used to design dumbbell shape geometry with X × Y × Z dimensions of 60 mm × 30 mm × 3 mm, respectively. This is because simple shape geometry can limit the multi-functionality and realization of the final configuration due to self-collision or contact [18]. The designed CAD model was further sliced using NewCreatorK (version 1.57.71) and uploaded to the 3D bioprinter for further printing process.

### 2.4. Temperature-Responsive Shape Memory Programming

**Figure 1:**
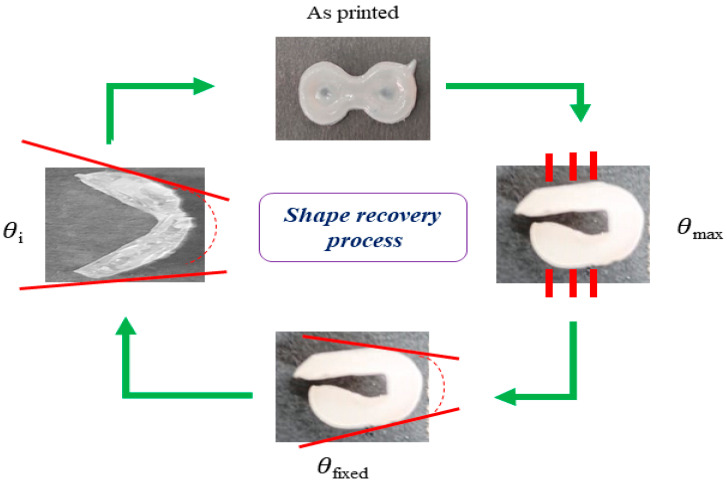
Schematic presentation of shape fixity and shape recovery process: *θ_max_* (maximum bending angle), *θ_fixed_* (Fixed angle), and *θ_i_* (Recovery angle).

Temperature-responsive shape memory programming of all printed construct (P1–P6) was conducted as per the earlier procedure mentioned [16]. Briefly, the printed dumbbell shape geometry was heated at 50 °C and was folded manually to provide a temporary shape. The maximum bending angle was measured (θ*_max_*). The modified shape was kept in a freezer at 4 °C under constant external force for 1 h to fix the folded shape. After 1 h the force and sample were removed and kept at room temperature until it was fully fixed. The fixed bending angle was denoted by θ*_fixed_*. Shape recovery was achieved by immersing a temporary shape in a water bath at 37 °C, and the test was conducted until a full shape recovery was observed. The recovered bending angle (θ*_i_*) was measured every 30 s. The shape fixity ratio and shape recovery ratio was determined using the following formulae [19] and further used for the development and validation of various ML models. The process is shown in Figure 1.

(1) $$Shape\;fixity\;ratio=\left(\frac{\theta_{fixed\;}}{\theta_{max}}\right)\times 100$$

(2) $$Shape\;recovery\;ratio=\left\{\frac{\theta_{max}-\theta_{i}}{\theta_{max}}\right\}\times 100$$

### 2.5. ML Modeling

#### 2.5.1. Data Splitting Strategy

To investigate the effect of PCL and p(NIPAM-4-ABP) concentrations (%*w*/*w*) on the shape recovery ratio, ML models were established, and the used dataset was divided into a training subset (85%) and a test subset (15%). The training subset was used for constructing models and tuning model hyper-parameters, and the test subset was used to evaluate the model prediction performance on unknown data. In the present study, a tenfold cross-validation method was used for tuning the model hyper-parameters to ensure the generalization ability of ML models [20].

#### 2.5.2. Construction of ML Models

To predict the shape recovery ratio, two ML algorithms were applied to construct regression models, including DT and MLR. Both models were constructed using the scikit-learn package in Python. For DT, the maximum depth of the tree and the minimum number of samples required to split an internal node were set to 2 and 2, respectively.

#### 2.5.3. Model Performance Criterion

In ML modeling, multiple evaluation metrics were adopted to assess the model performance for regression tasks. In this work, coefficient of determination (R^2^), MAE, and RMSE were selected to evaluate the model predictive accuracy for shape recovery ratio. These metrics were defined using the following Equations (3)–(5).

(3) $$R^{2}=\sum_{i=1}^{n}{\left(x_{i}-\;\bar{y}\right)}^{2}/\sum_{i=1}^{n}{\left(y_{i}-\;\bar{y}\right)}^{2}\;$$

(4) $$\mathrm{MAE}=(\sum_{i=1}^{n}\left|y_{i}-x_{i}\right|)/n$$

(5) $$\mathrm{RMSE}=\sqrt{\sum_{i=1}^{n}{\left(y_{i-}x_{i}\right)}^{2}/n}$$

where y_i_ = experimental value, x_i_ = predicted value, $\bar{y}$ = the mean of experimental values, and n = total number of samples.

#### 2.5.4. Assessment of Optimized Batch

The MLR equation of shape recovery was used to evaluate the optimized formulation.

### 2.6. Rheology Measurement

The shear-strain dependent rheology of both the optimized placebo and PCM-loaded gel was determined by parallel plate rheometer (MCR 302e, Anton Paar companies, Hellmuth-Hirth-Strasse, Ostfildern-Scharnhausen, Germany). The whole experiment was conducted at 25 °C. An amplitude sweep test was performed, which evaluates the reaction of the material to loading with increasing amplitude. The amplitude was increased from strain 0.0001 to 10 rad. Amplitude sweep shows where the storage modulus is almost the same (linear viscoelastic range), and sudden fall in storage modulus shows a non-linear viscoelastic region.

### 2.7. Raman Spectroscopy

Raman spectra of pure p(NIPAM-4ABP), PCM, BIS, optimized placebo 3D construct (P6), and optimized PCM-loaded 3D printed construct were recorded with a Horiba LabRAM HR Evolution, combined with an Olympus confocal microscope (HORIBA France SAS, Longjumeau, France). Various spectra were recorded in the range of 500–3200 cm^−1^, with an acquisition time of 10 s and a cumulative time of 5 s using an automatic baseline correction. The sincerity sensor was used with an objective magnification of 10, a grid of 1800 gr/mm, and 100% yarn with a wavelength of 532 nm laser irradiation (100% laser intensity).

### 2.8. DSC

DSC analysis of pristine p(NIPAM-4ABP), PCM, 4-ABP, NIPAM, P6, and optimized PCM-loaded 3D construct was performed using a DSC-3 STARe system (Mettler Toledo, Im Langacher 44, 8606 Greifensee Schweiz, Switzerland). All of the above samples were accurately weighed into an aluminum sample holder and hermetically sealed, as mentioned earlier. Thermograms were taken from room temperature to 300 °C at a heating rate of 10 °C/min under a flow of nitrogen gas at 20 mL/min to provide an inert atmosphere.

### 2.9. TGA

The thermal stability of p(NIPAM-4-ABP), PCM, 4-ABP, NIPAM, P6, and optimized PCM-loaded 3D construct was evaluated by TGA analysis using a TGA-2 STARe system (Mettler Toledo, Im Langacher 44, 8606 Greifensee Schweiz, Switzerland). The above samples were accurately weighed between 4–6 mg into aluminum oxide crucibles. The TGA profile was recorded from room temperature to 600 °C at a heating rate of 10 °C/min under a flow of nitrogen gas at 40 mL/min to provide an inert atmosphere.

### 2.10. XRD

X-ray diffractograms of p(NIPAM-4ABP), PCM, P6, and optimized PCM-loaded 3D construct were recorded using an X-ray diffractometer (Bruker AXS D8 Advance, Dynamostraße, Mannheim, Germany) coupled with 2θ (λ = 1.5406 Å), materials were positioned onto the sample vessel, and XRD diffractograms were taken with a 2θ plane with an angle range of 30–80° [21,22] with a scan speed of 10/min.

### 2.11. SEM

SEM analysis of the prepared P6, programmed 3D construct, and recovered 3D construct were analyzed using FESEM (JEOL JSM-7610F, Silver court watchmead, Welwyn Garden City, UK). The respective samples under examination were mounted onto double-sided NEM tape (NEM Tape, Nisshin EM. Co., Ltd., Tokyo, Japan), and sputtered with platinum for 2 min before imaging using an auto fine coater (JEOL JEC-3000FC, Silver court watchmead, Welwyn Garden City, UK). SEM micrographs images were acquired at the requisite magnification to capture desired acquisition.

### 2.12. Percent Entrapment Efficiency

The semi-solid gel (P6) containing 500 mg of PCM was extruded using SSE-based 3D printing. The 3D printed construct equivalent weight to 260.50 ± 2.25 mg was dissolved in 35 mL of methanol with constant sonication to break down the complete polymeric network. The mixture was placed in an incubator shaker (CIS-18 Plus, Remi Instruments Pvt. Ltd., Mumbai, India) for 24 h at 37 ± 0.5 °C at 100 rpm. The supernatant (1.0 mL) was analyzed for the estimation of PCM entrapment from 3D printed construct using a double-beam UV-visible spectrophotometer (UV2600, Shimadzu, Japan) at a wavelength of 245 nm.

### 2.13. In Vitro PCM Release Study

The *in vitro* release study of PCM from PCM-loaded temperature-responsive self-folding programmed 3D construct was conducted using a USP type II paddle apparatus (DS8000, LabIndia Pvt. Ltd., Mumbai, India) at a wavelength of 245 nm using a double-beam UV-visible spectrophotometer (UV2600, Shimadzu, Japan). Briefly, a 600 mL of 0.1 N HCL was taken as a gastric dissolution medium. The rpm was set at a speed of 75 rpm with a temperature set value of 37 °C (±0.5 °C), and *in vitro* sink conditions were kept throughout the point of study. An amount of 5.0 mL of sample aliquots was withdrawn at fixed time intervals, filtered to remove printed matrix debris, and further analyzed using the UV-visible spectrophotometer. The *in vitro* PCM release studies were performed in triplicate, and the cumulative amount of PCM released was determined as a function of time [23].

### 2.14. PCM Release Kinetic Study

The *in vitro* release profile was used to determine the mechanism of PCM release from programmed 4D construct. The PCM release data were evaluated for zero-order, first-order, Higuchi and Korsmeyer–Peppas drug release kinetic models using the DD solver Excel Add-in tool. The R^2^ and n values were used to investigate various release mechanisms.

## 3. Results and Discussion

### 3.1. SSE of 3D Construct

The SSE printer employed in this study used a syringe dispenser attached to 0.4 mm size dispensing nozzle. The step motor is used to dispense the gel mass loaded into the syringe through the nozzle to obtain a 3D construct. A trial-and-error approach was used to select the various printing parameters. The optimized printing parameters are listed in Table 2. In particular, the printing temperature and bed temperature were set at 27 °C and 4 °C, respectively, and the printing speed was chosen as 8 mm/s.

**Figure 2:**
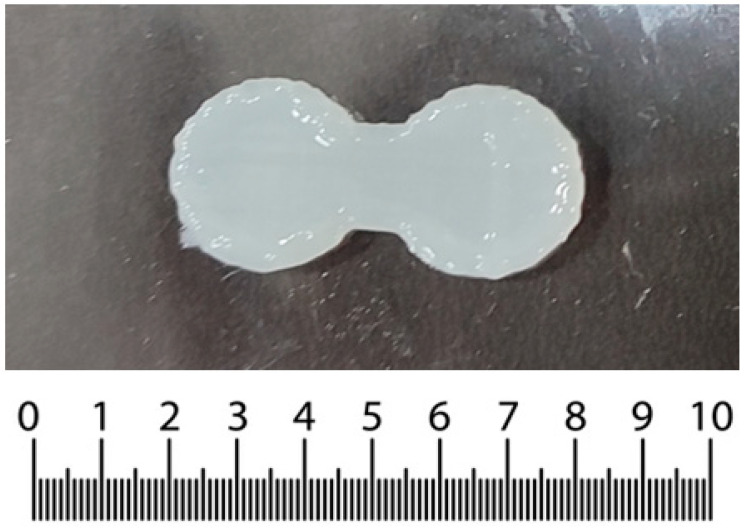
SSE-mediated 3D printed construct. Unit: mm.

The travel speed (the speed at which the bed moved in the x-y direction) was kept the same as the printing speed (the speed at which the gel was extruded) to avoid over-deposition or under-deposition of the gel. Further, the retraction speed (the speed at which the printer retracts the extruding gel mass while changing the printing position) and the retraction distance were kept at 1 mm/s and 1 mm, respectively. The 3D construct for further studies was printed using the above-mentioned optimized printing parameters (Figure 2).

### 3.2. Temperature-Responsive Shape Recovery

Shape recovery of the printed 3D construct was performed as per the procedure mentioned in our previous work [16]. All the 3D-printed constructs were programmed into a temporary shape. Afterward, the samples were immersed in a water bath, which was previously maintained at 37 °C to achieve shape recovery. The recovery angle was measured using ImageJ software. The test was conducted in sextuplicate for reproducibility. A 93% shape recovery was observed when the concentration of p(NIPAM-4-ABP) was 100%, while the decrease in shape recovery ratio was observed as the concentration of p(NIPAM-4-ABP) decreased (Supplementary Figure S1). Furthermore, the shape fixity ratio was calculated according to Equation (1), and it was found that the concentration of p(NIPAM-4-ABP) had an influence on the shape fixity of the temporary programmed shape (Supplementary Figure S2). In the case of 100% p(NIPAM-4-ABP), the shape fixity ratio was stabilized at 90.07 ± 4.30% after 30 min and remained unchanged until shape recovery was initiated.

### 3.3. Comparison of Different ML Models

**Figure 3:**
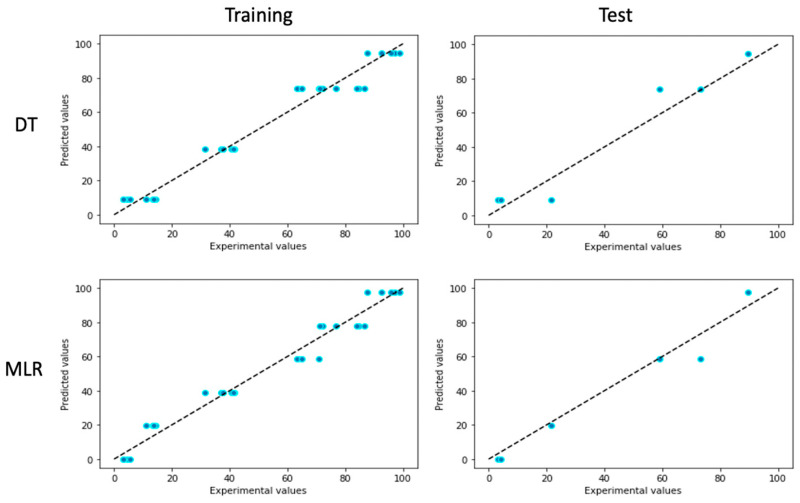
Scatter plots of predicted values calculated by DT (**top**) and MLR (**below**) models vs. experimental values on the training and test subset.

To predict the shape recovery ratio, two ML models, DT and MLR, were established and compared. R^2^, MAE, and RMSE were employed to evaluate the model performance (Table 3). R^2^ indicates the proportion of variance in the response variable that is explained by the independent variable(s) [24]. MAE, the average absolute difference between the predicted value and the observed value, is used to measure how far predicted values are from the actual observations [25]. In regression analysis, RMSE is the standard deviation of the residual, an error between the predicted value and the observed actual value [26]. Based on the calculated result, MLR shows the optimal predictive performance, with higher R^2^ and smaller MAE and RMSE compared to the DT model. The scatter plots in Figure 3 exhibited the predicted values calculated by MLR and DT versus the experimental values on the training and test subset.

### 3.4. Assessment of Optimized Batch

In addition to the superior predictive performance, the MLR model equation can be used to determine the optimal batch from P1–P6, with a predetermined optimal shape recovery and shape fixity ratio. The obtained MLR equation is listed below:

(6) $$y=\left(-0.486\times x1\right)+\left(0.486\times x2\right)+48.812\;$$

where *x*1 refers to PCL concentration (%*w*/*w*), *x*2 refers to p(NIPAM-4-ABP) concentration (%*w*/*w*), and *y* refers to shape recovery ratio. The shape recovery ratio was calculated using obtained MLR equation for each batch (Table 4).

Hence, based on the obtained values for shape recovery ratio, the batch showing the highest value of shape recovery ratio could be considered as the optimal batch for further studies. The obtained data conveys that batch P6 showed the highest shape recovery property and is considered as an optimized batch for further studies.

### 3.5. Rheology Measurement

The result of the amplitude sweep test was presented as shear strain ϒ (%) on the x-axis and storage modulus (G′) and loss modulus (G″) on the y-axis (Supplementary Figure S3). Further, based on the obtained data for G′ and G″, the linear viscoelastic range (LVR) is determined. The LVR is the range in which the test can be carried out without destroying the sample structure, also known as the linearity limit. In the LVR region, the values of G′ and G″ are constant. Based on the data, the linearity limit of the LVR region for placebo and PCM-loaded gel are 1322.8 Pa (0.0118%) and 1600.1 Pa (0.0118%), respectively. Moreover, the values of G′ and G″ in the LVR region are also assessed to predict the viscoelastic nature of the sample. The data shows that the storage modulus (G′) values are more significant than the loss modulus (G″); this indicates solid viscoelastic material. In both cases, the curve of G′ drops constantly after leaving the LVR region, which means a slow breakdown of both gels. The obtained results confirm that the addition of PCM cannot contribute to the mechanical properties of the material, possessing good extrudability during semisolid extrusion 3D printing.

### 3.6. SSE of Optimized Formulation

The SSE of the optimized batch in terms of shape recovery and shape fixity ratio was performed, as discussed previously in Section 3.1 (Supplementary movie).

### 3.7. Raman Analysis

Raman spectra of p(NIPAM-4ABP), PCM, BIS, P6 and PCM-loaded 3D printed constructs were obtained for the chemical analysis. The Raman spectra of PNIPAM-4ABP (Supplementary Figure S4a), in particular, the stretching bands in 2850–3000 cm^−1^, is associated with the C-H and CH_2_ moiety in the isopropyl group involving amide functionality. The highest frequency of CH antisymmetric stretching (2977 cm^−1^) is due to the greater number of water molecules surrounding the CH group [27]. The shifting of this peak could be correlated with the temperature-responsive hydration and dehydration of the isopropyl moiety in the PNIPAM-4ABP network. Further, the peaks at 849, 948, 1160, 1320, and 1653 cm^−1^ correspond to the C-C stretching, C-C skeletal stretching, C-O-C stretching, C-N stretching, and amide stretching, respectively. In addition, the intensity ratio between symmetric and antisymmetric peaks at 2881, 2925, and 2977 cm^−1^ are usually related to the lateral packing density of the polymer chain, and any changes in that convey polymer/polymer or polymer/drug interaction [27]. Further, the Raman spectra of pure PCM (Supplementary Figure S4b) is recognizable compared to the available literature [28]. The presence of particular bands such as 797, 858, and 1237 cm^−1^ represents CNC ring stretching, ring breathing, and C-C ring stretching, respectively. Further, the Raman spectra of BIS (Supplementary Figure S4c) show a strong peak at 1631 cm^−1^ corresponding to the C=O stretching. The C-N stretching vibration (amide III) is observed near 1326 cm^−1^. The C-C bending vibrations around 960 cm^−1^ can be seen in the given spectra. The bands around 2964 to 3098 cm^−1^ represent C-H stretching vibration and CH_2_ asymmetric stretching vibration. In addition, Raman spectra of the both placebo and PCM-loaded 3D construct (Supplementary Figure S4d) showed all the bands of PNIPAM-4ABP with some characteristic bands of PCM and BIS. Interestingly, the peak intensity between the 2800–3000 cm^−1^ region shows a significant difference. The observed difference could be due to the involvement of CH_3_ and CH_2_ groups in the isopropyl moiety of PNIPAM-4ABP for the formulation of the hydrogel.

### 3.8. DSC Analysis

**Figure 4:**
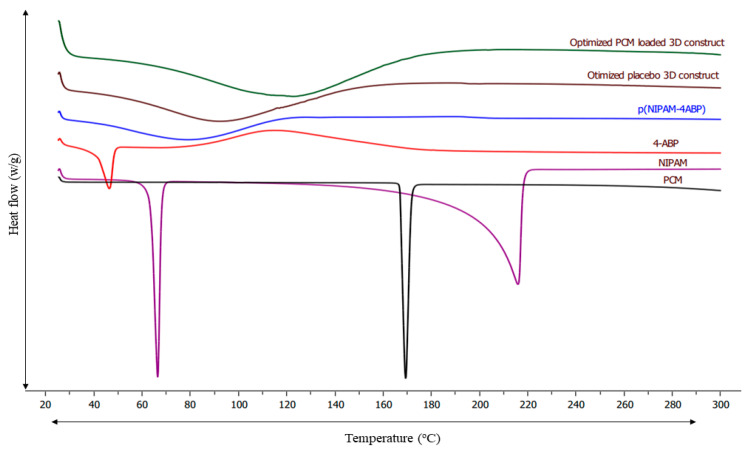
DSC thermogram of p(NIPAM-4ABP), PCM, 4-ABP, NIPAM, optimized placebo 3D construct, and optimized PCM-loaded 3D construct.

The DSC study was conducted to predict the thermal signature of p(NIPAM-4ABP), PCM, 4-ABP, NIPAM, P6, and optimized PCM-loaded 3D construct. In addition, the study was conducted to determine their physicochemical properties. The DSC thermogram of all samples is shown in Figure 4. The thermogram of pure PCM displayed a sharp endothermic peak near 170 °C with enthalpy of fusion of −211.13 J/g, which attributes to the crystalline nature of the PCM. The thermogram of pure NIPAM showed a sharp peak near 65 °C representing the melting transition of NIPAM near this temperature. Furthermore, the thermogram of 4-ABP also showed the melting transition near 45 °C. The thermogram of pure p(NIPAM-4-ABP) shows a broad endothermic peak starting at 40 °C and ending at 80 °C, with enthalpy of fusion of −372.01 J/g. This endothermic transition may be due to the hydrophilic hydration of the amide group and the hydrophobic hydration of the isopropyl group [29]. Further, the DSC thermogram of the p(NIPAM-4-ABP)-PCM 3D construct showed the same endothermic transition as pure p(NIPAM-4-ABP), with the enthalpy of fusion of −584.32 J/g. This analysis confirms that there is some level of compatibility with the p(NIPAM-4-ABP)-PCM system due to reduction in the enthalpy of fusion [30]. In addition, the absence of a PCM endotherm in the PCM-loaded construct showed amorphous behavior of PCM in the p(NIPAM-4-ABP) system.

### 3.9. TGA Analysis

**Figure 5:**
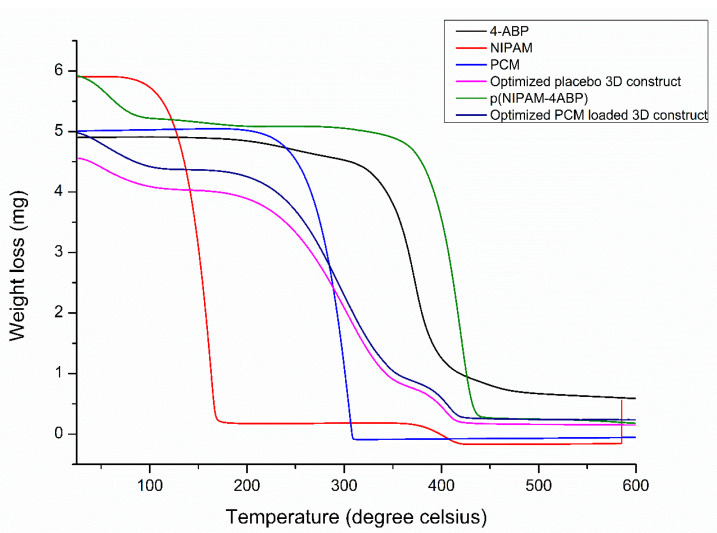
TGA profile of p(NIPAM-4ABP), PCM, 4-ABP, NIPAM, optimized placebo 3D construct, and optimized PCM-loaded 3D construct.

Figure 5 revealed the TGA profile of p(NIPAM-4ABP), PCM, 4-ABP, NIPAM, P6, and optimized PCM-loaded 3D construct. The TGA profile of p(NIPAM-4ABP) verified the successful modification of the PNIPAM network with 4-ABP. In the TGA curve of pure PCM, a weight loss of around 84% was observed in the region of 210–310 °C. Before this region, no weight loss was observed, revealing the thermal stability of PCM. In the curve of 4-ABP, no degradation was observed near its melting point. The % weight loss of around 86% was observed in the region of 321–500 °C. Further, the TGA curve of p(NIPAM-4ABP) showed weight loss near 90 °C attributed to the loss of water molecules. The extent of weight loss was observed in the region of 351–450 °C. This weight loss is due to the breakdown of the polymer chain backbone. In addition, the TGA curve of p(NIPAM-4ABP) showed an improvement in the thermal stability of pure NIPAM. The TGA curves of the placebo and PCM-loaded 3D construct showed identical thermal signatures. In both cases, a slight weight loss of around 100 °C was observed. This could be due to the loss of bound water. The continuous decline in % weight was observed for both the curves in the region of 220–420 °C. This decline could be due to the degradation of the polymeric backbone of p(NIPAM-4ABP) gel. Overall, the results convey that pure p(NIPAM-4ABP) has identical thermal stability with placebo and drug-loaded 3D constructs. In addition, the obtained results showed improvement in the thermal stability of pure NIPAM after chemical modification.

### 3.10. XRD Analysis

**Figure 6:**
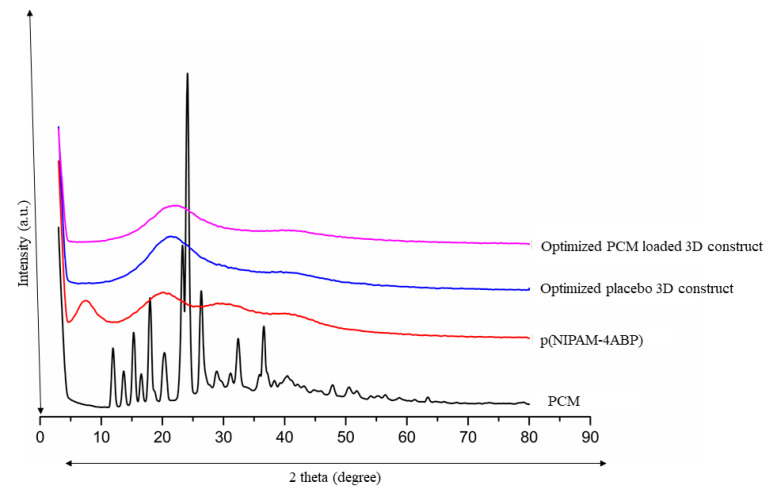
XRD pattern of p(NIPAM-4ABP), PCM, optimized placebo 3D construct, and optimized PCM-loaded 3D construct.

XRD diffractograms of p(NIPAM-4ABP), PCM, P6, and optimized PCM-loaded 3D construct are shown in Figure 6. XRD data of pure PCM show some characteristics of intense diffraction peaks at 2θ of 15°, 25°, and 28° which reveals the crystalline nature of pure PCM. The obtained diffraction peaks are similar with the previously available literature [21]. In addition, the XRD diffractogram of p(NIPAM-4-ABP) showed two wide diffraction peaks, one at 2θ of around 10° and the second at 20°. The obtained wide angle could not clearly define reflections and showed a lower degree of crystallinity. Furthermore, the XRD diffractogram of the placebo and PCM-loaded construct showed a pattern similar to pure p(NIPAM-4-ABP), which proves the amorphization of PCM in a polymer matrix.

### 3.11. SEM

**Figure 7:**
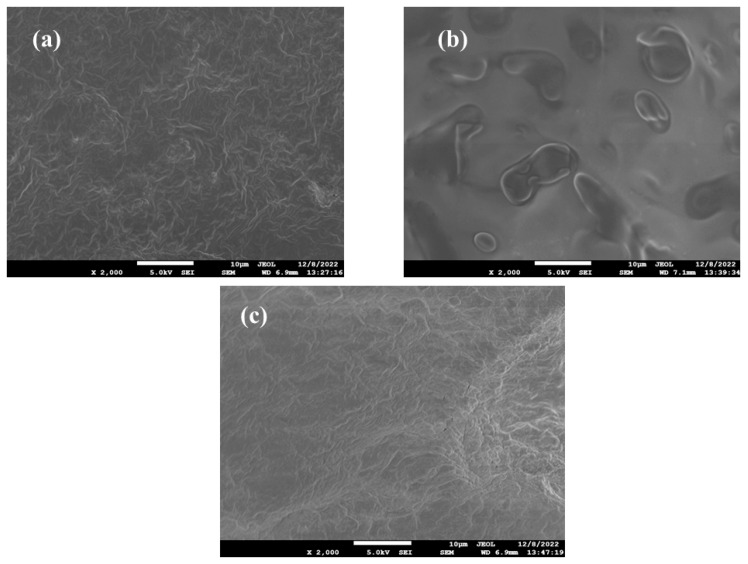
SEM images of (**a**) optimized placebo 3D construct without programming, (**b**) temporary shape, (**c**) recovered 3D construct.

The surface morphological properties of PCM-loaded p(NIPAM-ABP) 3D constructs before and after shape memory programming were assessed using SEM. Figure 7 shows the representative images of the 3D construct before and after the shape memory programming. The SEM image of the 3D construct without any programming Figure 7a conveys the uniform crosslinking of the polymeric network and has a relatively smooth surface. The heating of the 3D construct above the glass transition temperature can result in the deformation of the polymer chain due to the high mobility of the polymeric chain at this temperature. It could be fixed by lowering the temperature below its glass transition temperature by limiting the mobility of the polymeric chain [31]. The SEM image of the temporary shape (heating followed by freezing at −20 °C) shows the formation of large channels in the polymeric chain, which shows the deformability of the polymeric chain above the glass transition temperature (Figure 7b). The recovery process of the polymeric chain is initiated by subjecting the prototype above the glass transition temperature; the polymer chain regains its mobility and recovers to its original state. The SEM image of the recovered prototype (Figure 7c) visually confirms the nearly original state of the polymeric chain.

### 3.12. Percent Entrapment Efficiency

The % PCM in the 3D construct was evaluated using the UV-visible method. The % entrapment efficiency of PCM in the 3D printed construct was found to be 98.11 ± 1.5%. The obtained data from the % PCM assay are acceptable and indicate homogeneous distribution of PCM in optimized batch with excellent entrapment behavior.

### 3.13. In Vitro PCM Release Study

**Figure 8:**
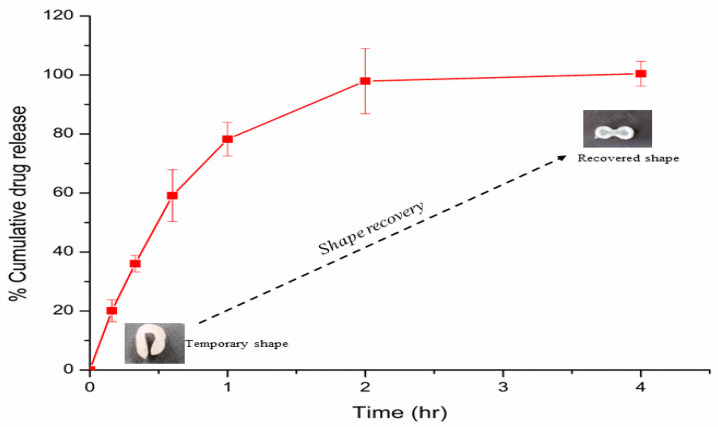
*In vitro* release behavior of PCM from the programmed (temporary shape) 3D printed construct at pH 1.2 in 0.1 N HCl.

The dissolution profile of the PCM-loaded 3D construct after programming it into a temporary state was kept in gastric pH condition. The complete release of PCM was noted within 4 h, as shown in Figure 8. The average percentage of cumulative PCM dissolved within 4 h was approximately 100 ± 4.19%. The *in vitro* release data showed the biphasic release pattern of PCM from the PCM-loaded 3D construct. In detail, immediate release of around 75% was observed within 1 h, followed by the sustained release pattern, attaining 100% release within 4.0 h. Further, the release profile of PCM showed 100% release within 2 h, showing suitable matrix system for fasting state condition. Moreover, the release profile showed steady phase after complete drug release up to 4 h (maximum gastric emptying time), showing suitable matrix system for feed state condition. Further, the visual inspection of the dissolution process revealed complete shape recovery of programmed 3D construct during the dissolution study.

### 3.14. PCM Release Kinetic Study

The data obtained from the release kinetic models is listed in Table 5. The drug release data fitted well in the first-order kinetic model, indicating that the drug release mechanism is concentration-dependent. Thus, the PCM released from the 4D construct was a fraction of the remaining PCM in the p(NIPAM-4ABP) matrix. In addition, the goodness of fit (R^2^) for the Higuchi model was found to be 0.8832, indicating that the mechanism of PCM release from this matrix was diffusion controlled [32]. Further, to explore the release pattern of PCM, *in vitro* release data was fitted to the Korsmeyer–Peppas model. The model fits the data well and the best fit value for n was found to be 0.376. This implies that the PCM release from such a matrix is classical Fickian-type diffusion. The *in vitro* release data showed a biphasic release pattern (Figure 8), the first phase being related to the diffusion of the hydrophilic group, which implies initial burst release of the PCM. At a temperature of 37 °C, i.e., above the LCST, the concept of polymeric relaxation becomes predominant, approaching the second phase of drug release. The findings were in agreement with C.N. Hernandez-Tellez et al. [33]. Thus, based on the findings, it could be concluded that the process of PCM dissolution and water uptake by the 4D construct would occur during the release of PCM; this water uptake enhances the molecular mobility of p(NIPAM-4ABP) polymeric system, and causes chain relaxation that enables PCM release from p(NIPAM-4ABP) matrix at 37 °C.

## 4. Conclusions

In the present work, we explored the pharmaceutical application and drug release capabilities of a 4D printed construct as a potential delivery device. We evaluated the use of our synthesized temperature-responsive self-folding feedstock to develop a 4D printed construct as a drug delivery system. The synthesized feedstock was converted to a gel to evaluate its viscoelastic nature using rheological measurements. The obtained rheological data conveyed the viscoelastic nature of the synthesized gel and good extrudability during SSE-mediated 3D printing. Furthermore, we combined the synthesized gel with SSE 3D printing to print a 3D construct. The printed construct was systemically evaluated for the shape memory test. Parameters such as shape fixity and shape recovery ratio were used to train the ML/AI algorithm and to optimize the best formulation in terms of shape recovery ratio. Furthermore, the optimized batch was systematically evaluated to characterize *in vitro* physicochemical properties using various analytical tools. It was observed that the physicochemical evaluation of the optimized construct confirms the absence of physicochemical interaction between placebo and PCM-loaded construct, exhibiting good thermal stability, including excellent entrapment behavior, with homogeneous distribution of PCM inside the 4D printed matrix. Finally, the *in vitro* release suggests 100% of PCM release from the programmed construct, showing the potential of 4D printing technology in constructing innovative smart drug delivery systems.

## Figures and Tables

**Table 1:** Study design for the preparation of placebo feedstock.

Code	P(NIPAM-4ABP) (%*w*/*w*)	PCL (%*w*/*w*)	Total Weight (%*w*/*w*)
P1	00	100	100.0
P2	20	80	
P3	40	60	
P4	60	40	
P5	80	20	
P6	100	00	

**Table 2:** Optimized printing parameters for the preparation of both placebo and PCM-loaded 3D printed construct.

Parameters	Optimized Value
Nozzle size	0.4 mm
Distance between nozzle tip and build plate	0.2 mm
Layer height	0.2 mm
Printing temperature	27.0 °C
Build-bed temperature	4.0 °C
Printing speed	8.0 mm/s
Travel speed	8.0 mm/s
Retraction speed	1.0 mm/s
Retraction distance	1.0 mm

**Table 3:** ML model performance of predicting shape recovery ratio.

ML Algorithms	Training Set			Test Set		
	R^2^	MAE	RMSE	R^2^	MAE	RMSE
MLR	0.97	4.89	5.66	0.957	5.26	7.05
DT	0.968	4.93	5.91	0.933	7.3	8.75

**Table 4:** Calculated values for shape recovery ratio using MLR equation.

Sample Code	P(NIPAM-4ABP) (%*w*/*w*)	PCL (%*w*/*w*)	Shape Recovery Ratio
P1	00	100	0.20
P2	20	80	19.64
P3	40	60	39.09
P4	60	40	58.53
P5	80	20	77.97
P6	100	00	97.41

**Table 5:** Goodness of fit values for different release kinetic models.

Release Kinetic Model	R^2^	n
Zero-order	0.2885	-
First-order	0.9979	-
Higuchi	0.8832	-
Korsmeyer–Peppas	0.9270	0.376

## Data Availability

The data presented in this study are available on request.

## Supplementary Materials

The following supporting information can be downloaded at: https://www.mdpi.com/article/10.3390/pharmaceutics15041266/s1.

## Abbreviations

4D	Four-Dimensional
ML	Machine Learning
SSE	Semi-solid extrusion
PCM	Paracetamol
PCL	Polycaprolactone
PNIPAM	poly(*N*-isopropyl acrylamide)
4-ABP	4-Acryloyloxybenzophenone
p(NIPAM-4-ABP)	poly(*N*-isopropyl acrylamide)-4-Acryloyloxybenzophenone
DSC	Differential Scanning Calorimetry
TGA	Thermogravimetric analysis
XRD	X-ray diffraction
SEM	Scanning electron microscopy
MAE	Mean absolute error
RMSE	Root means square error
MLR	Multiple linear regression
DT	Decision tree
