# Supplementary material for: Four-Dimensional Printed Construct from Temperature-Responsive Self-Folding Feedstock for Pharmaceutical Applications with Machine Learning Modeling
Source: Pharmaceutics. 2023 Apr 18;15(4):1266. doi: 10.3390/pharmaceutics15041266 (PMC10146263; doi:10.3390/pharmaceutics15041266)
Supplement: Supplementary file 1 [file pharmaceutics-15-01266-s001.zip › pharmaceutics-2336630-supplementary.pdf]

Supplementary Figures

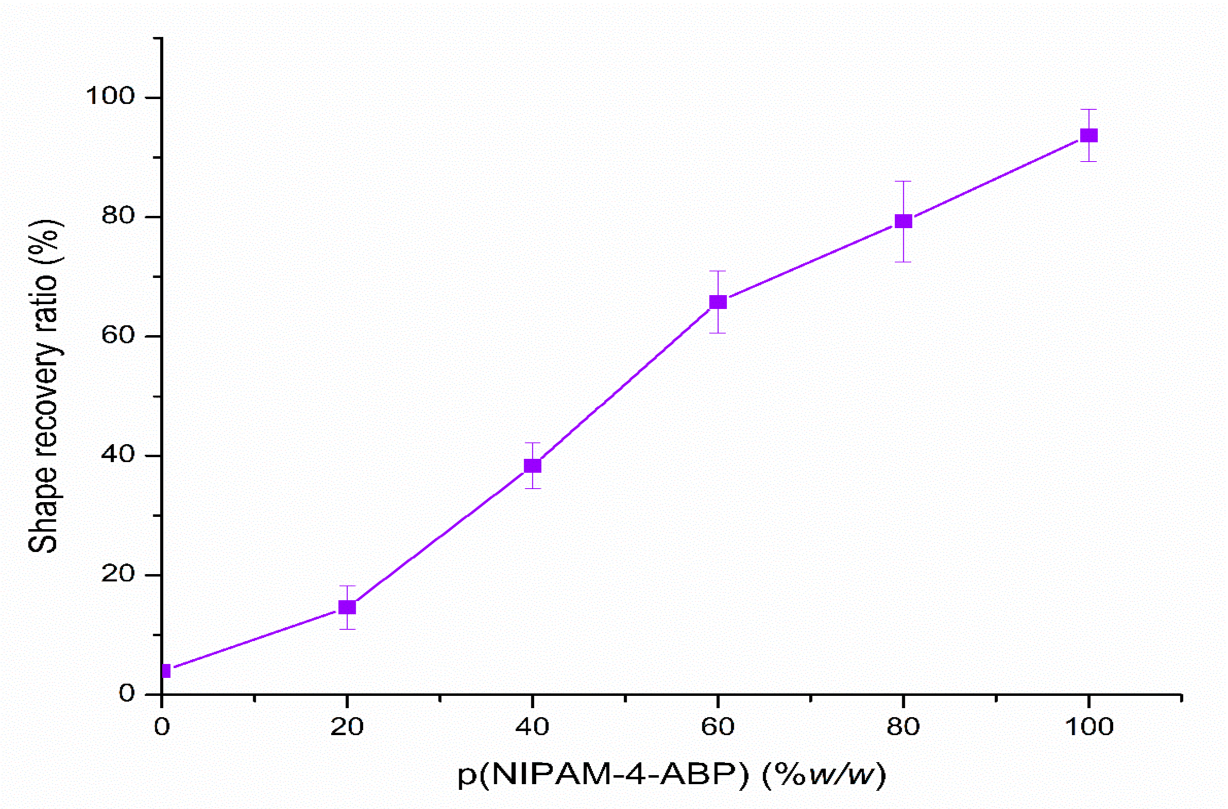

Supplementary Figure S1. Relation between shape recovery ratio (%) versus % w/w of p(NIPAM-4-ABP). The experiment was performed in n=6.

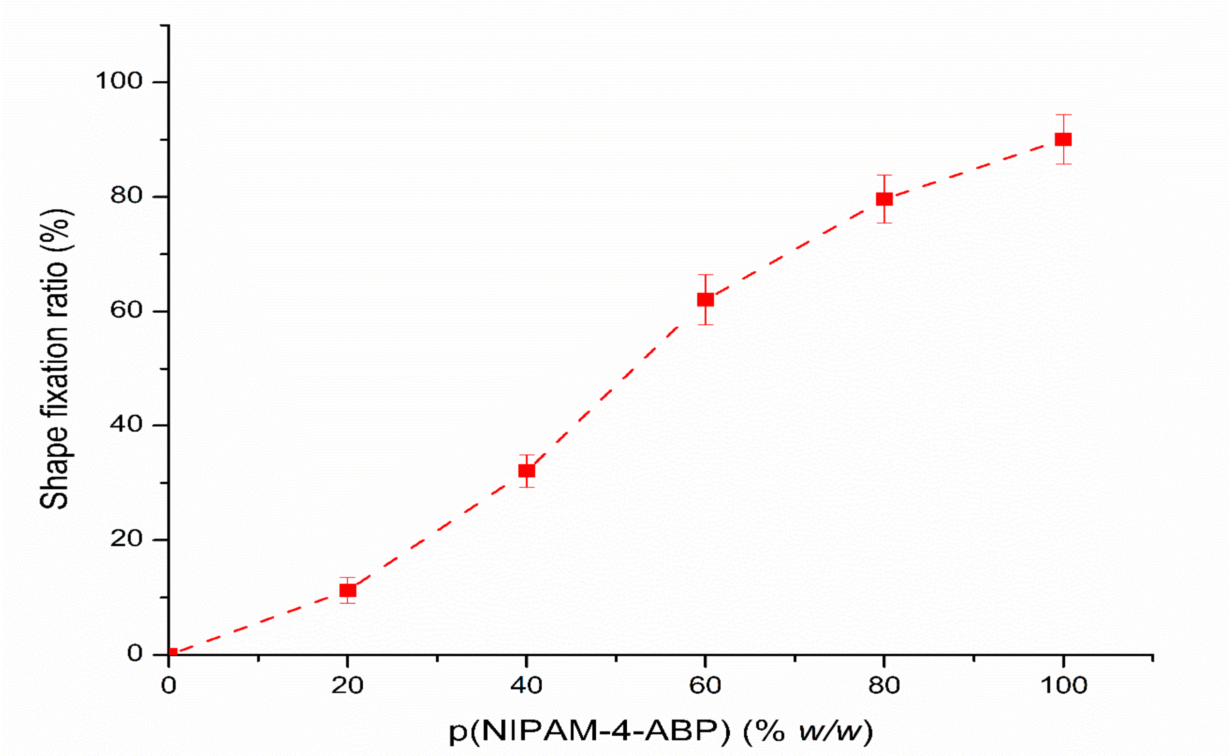

Supplementary Figure S2: Relation between shape fixation ratio (%) versus % w/w of p(NIPAM-4-ABP). The experiment was performed in n=6.

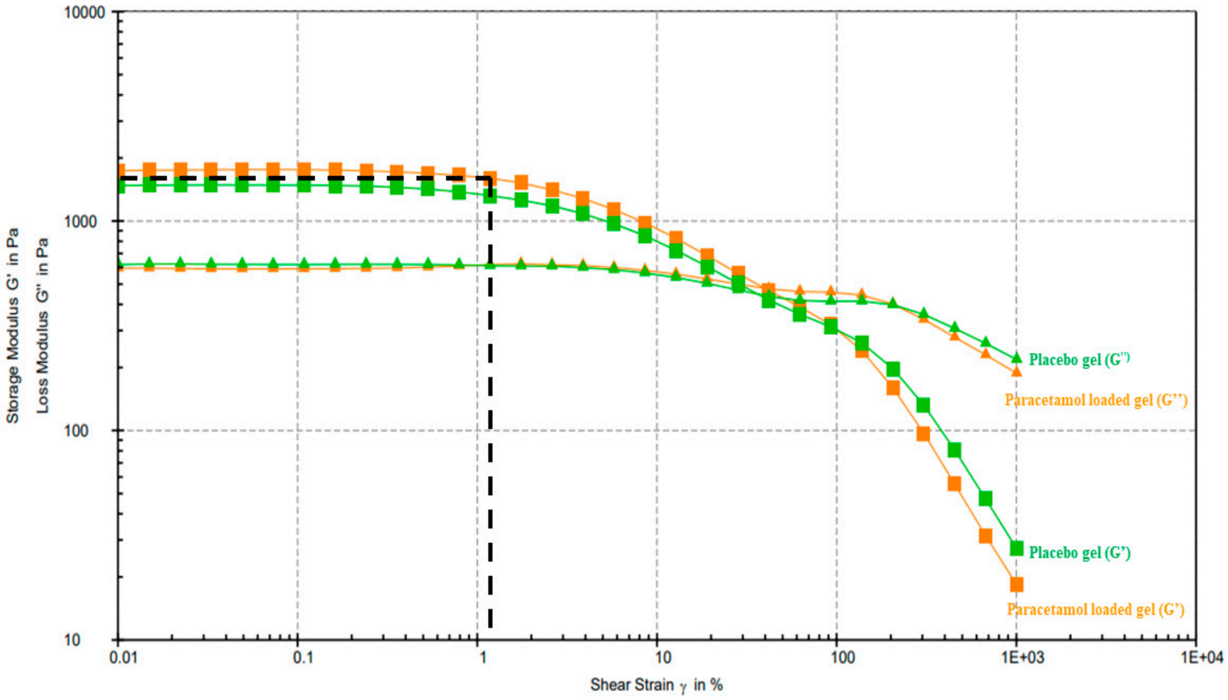

Supplementary Figure S3: Amplitude sweep test as a function of storage modulus (G') and Loss modulus (G''). G'> G'' sample has solid viscoelastic behaviour.

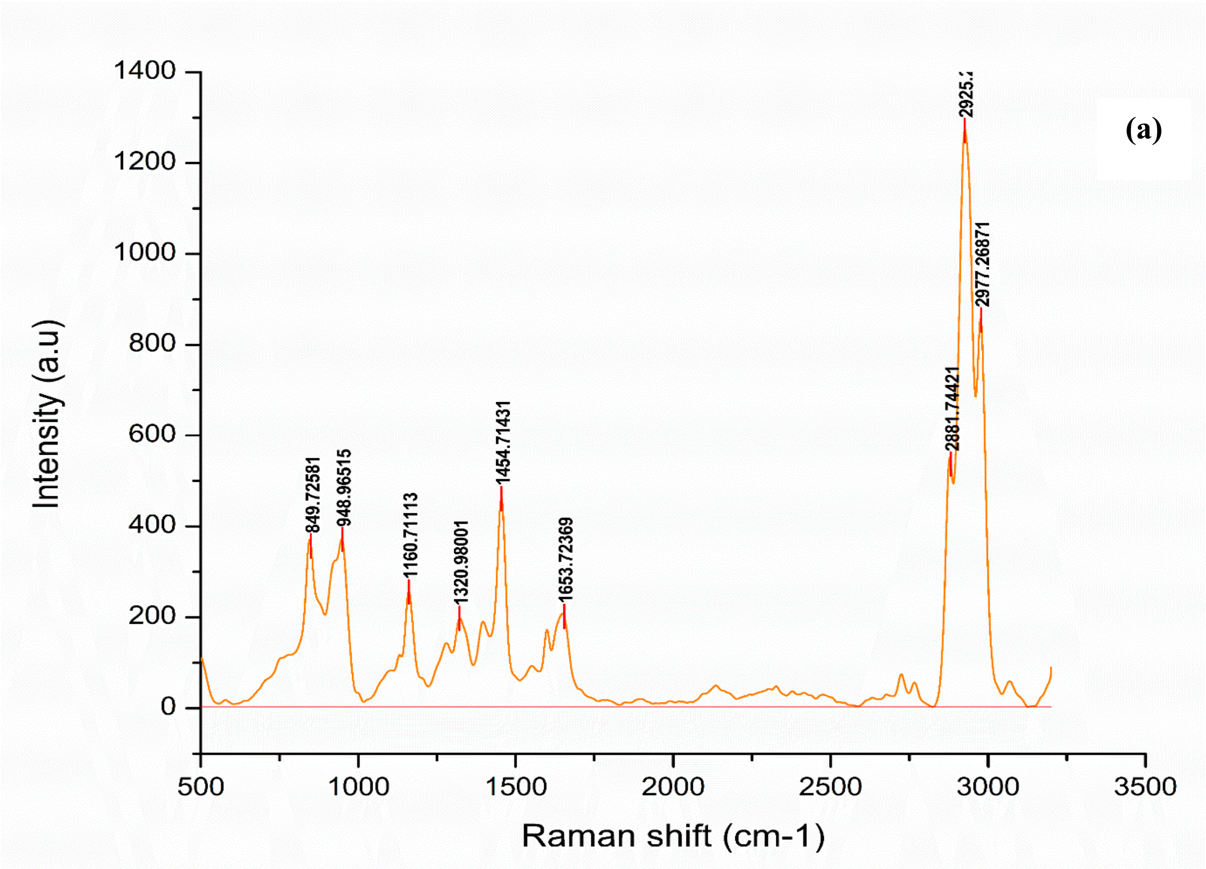

(a)
Intensity (a.u)
Raman shift (cm-1)
0
200
400
600
800
1000
1200
1400
500
1000
1500
2000
2500
3000
3500
849.72581
948.96515
1160.71113
1320.98001
1454.71431
1653.72369
2881.74421
2925.
2977.26871

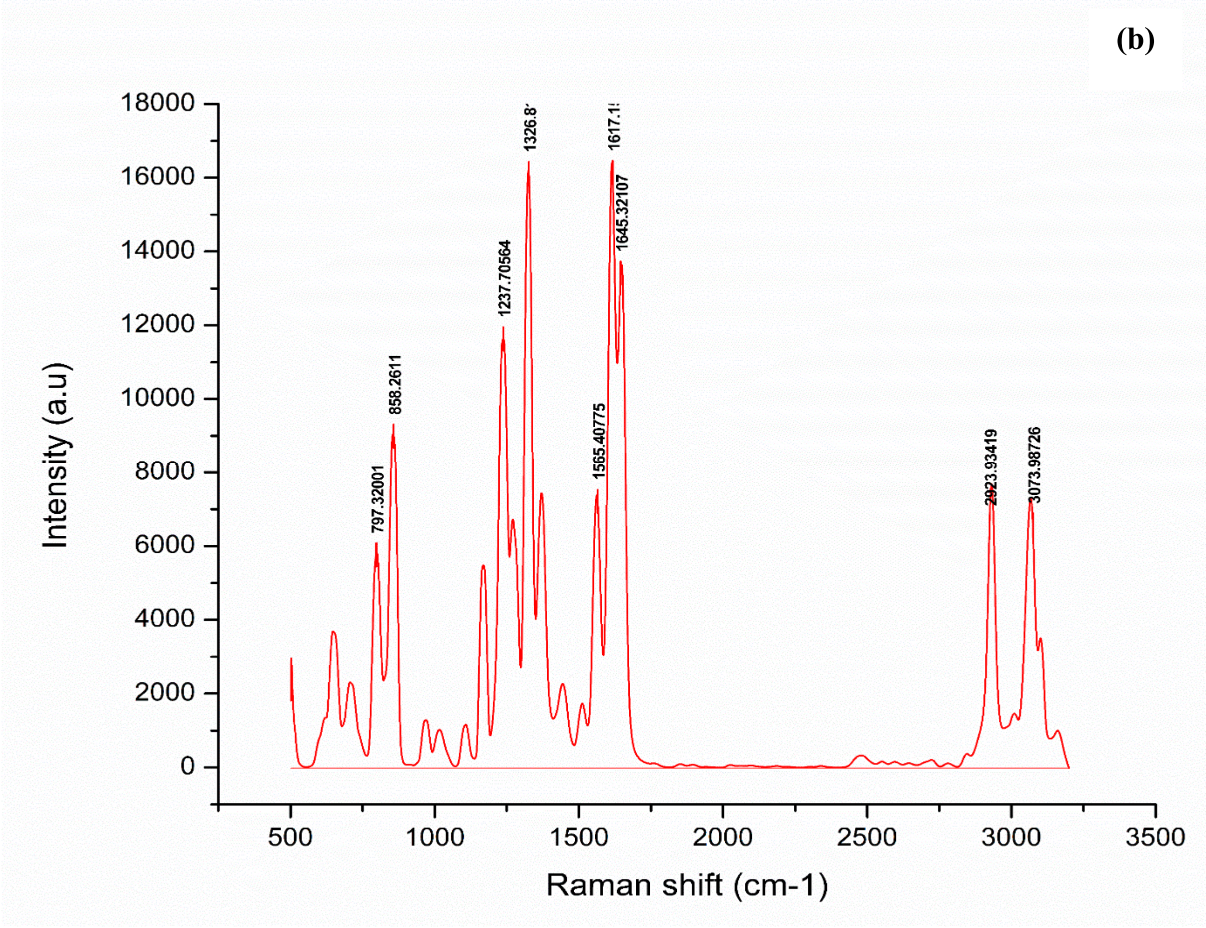

(b)
Intensity (a.u)
Raman shift (cm-1)
0
2000
4000
6000
8000
10000
12000
14000
16000
18000
500
1000
1500
2000
2500
3000
3500
797.32001
858.2611
1237.70564
1326.8
1565.40775
1617.1
1645.32107
2923.93419
3073.98726

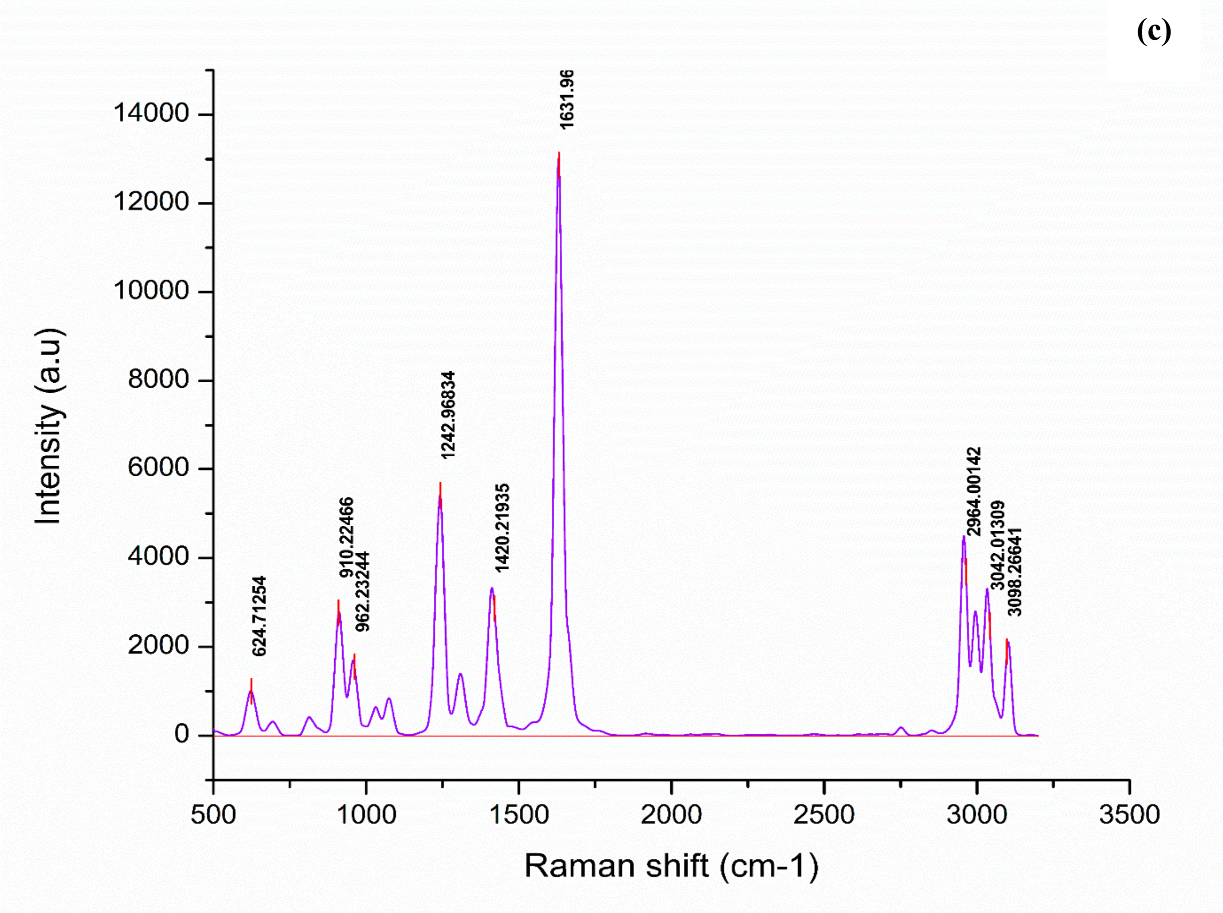

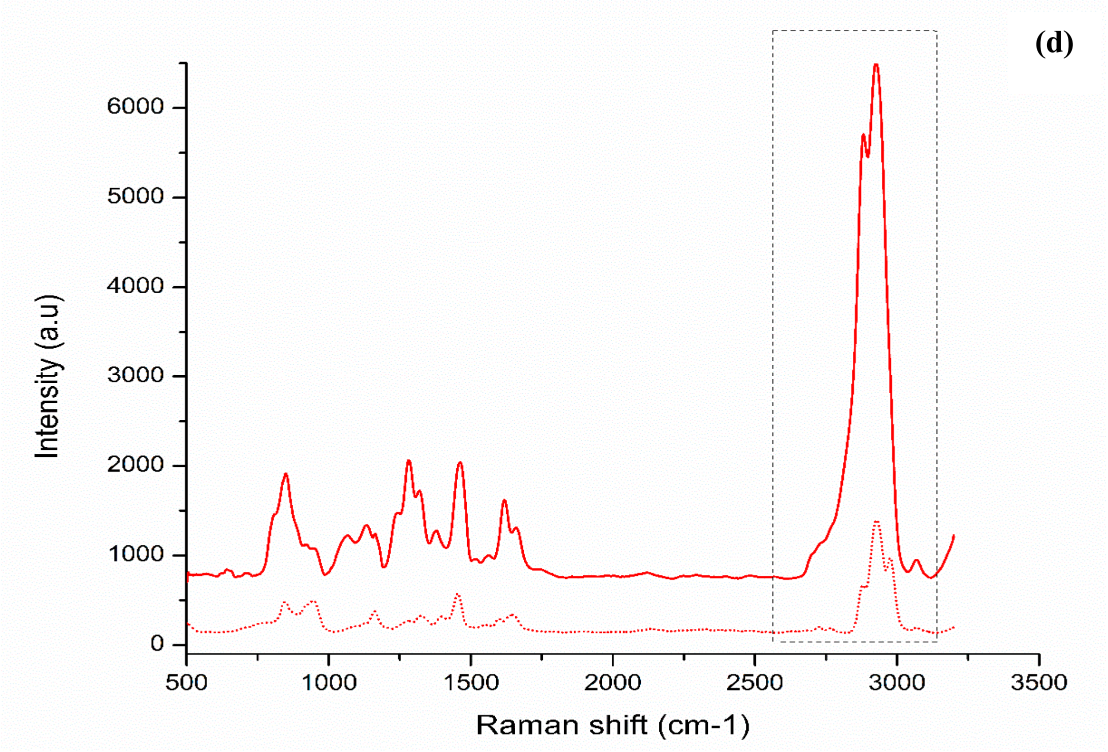

Supplementary Figure S4: Raman spectra of **(a)** p(NIPAM-4ABP), **(b)** PCM, **(c)** BIS, **(d)** optimized p(NIPAM-4ABP) placebo (⋯⋯) and PCM-loaded 3D construct (——).
